# Circulating miR-146a expression as a non-invasive predictive biomarker for acute lymphoblastic leukemia

**DOI:** 10.1038/s41598-021-02257-4

**Published:** 2021-11-23

**Authors:** Samiah Shahid, Wajeehah Shahid, Jawaria Shaheen, M. Waheed Akhtar, Saima Sadaf

**Affiliations:** 1 School of Biochemistry and Biotechnology, University of the Punjab, Lahore, 54590 Pakistan; 2 Institute of Molecular Biology & Biotechnology, The University of Lahore, Lahore, Pakistan; 3 Department of Physics, The University of Lahore, Lahore, Pakistan; 4 School of Biological Sciences, University of the Punjab, Lahore, 54590 Pakistan

**Keywords:** Cancer, Molecular biology, Biomarkers, Diseases, Oncology

## Abstract

Dysregulation of non-coding microRNAs during the course of tumor development, invasion and/or progression to the distant organs, makes them a promising candidate marker for the diagnosis of cancer and associated malignancies. This exploratory study aims at evaluating the usefulness of plasma concentration of circulating mir-146a as a non-invasive biomarker for acute lymphoblastic leukemia (ALL). Total RNA including miRNA was isolated from 110 plasma samples of patients (*n* = 66), healthy controls (*n* = 24) and follow up (*n* = 20) cases and reverse transcribed. Relative concentrations were assessed using real-time quantitative PCR and fold-change was calculated by 2^−ΔΔCt^ method. Finally, relative concentrations were correlated to clinicopathological factors. Patients (*n* = 66) were analyzed to determine fold expression of miR-146a in plasma samples of ALL. Before chemotherapy, pediatric (*n* = 42) and adult (*n* = 24) showed overexpression of miR-146a compared with healthy controls (*P* < 0.0001). There was no effect of age and gender on mir-146a expression in plasma. mirR-146a expression was independent of clinical and hematological features. Moreover, miR-146a levels in plasma of paired samples (*n* = 20) after treatment showed significant decrease in expression (*P* < 0.001). Expression of plasma miR-146a may be utilized as non-invasive marker to diagnose and predict prognosis in pediatric and adult patients with ALL. Moreover predicted targets may be utilized for ALL therapy in future.

## Introduction

Acute lymphoblastic leukemia (ALL) is characterized by hematological malignancy resulting uncontrolled lymphoid lineage proliferation of the hematopoietic cells. It is a disease of bone marrow that has effect both on children and adults^1^. The peak incidence of ALL is between two to five years of age with higher ratio in male than female^2^. However, in adults, the worldwide incidence of ALL is approximately one in 100,000 per year^3^. A study reported an estimated 6250 new cases of ALL and 1450 deaths in 2015^4^, rendering it the most common cause of death in developed as well as developing countries like Pakistan. The diagnosis of ALL is usually based on the histopathological examination including bone marrow aspiration and biopsy supplementing with immunologic, cytogenetic and molecular characteristics^5^. Although current standard methods for diagnosis of ALL have greatly improved diagnosis, but their clinical applications are limited due to their unpleasant, invasive and inconvenient nature. It is, indeed, of great concern to develop such diagnostic procedures which overcome these limitations including invasive procedures of bone marrow aspiration and biopsy. Many efforts have been done in the past for developing diagnostic markers that can easily and precisely diagnose specific diseases and its progression. Most of these markers are protein in nature but these are not satisfactory for tumor diagnosis due to their low sensitivity and specificity for detection^6^. Recently, advanced improvements in diagnosis and treatment strategies have improved pediatric outcome of ALL in developed countries where survival rate has reached to about 90%^7^. However, in developing countries the rate of survival is significantly reduced, possibly due to population differences, poor economic status, late diagnosis, less availability of advanced therapeutics, toxic effects of chemotherapy and most importantly lack of clinical trials on account of diagnosis and prognosis^8^. Thus, non-invasive diagnosis and improved prognosis still remained a great challenge for research particularly in Pakistan. The discovery of non-coding RNAs called microRNAs (miRNAs) opened a new horizon to discover novel diagnostic and prognostic markers for several malignant disorders including leukemia^9^. Studies on expression profiling of miRNA have shown its role in the development of ALL^10–12^. A single miRNA can regulate the expression of more than one gene, playing role in different stages of hematopoiesis and deregulation at any level may lead to leukemia^10^. miRNAs help to establish accurate classification of leukemia and to determine origin of the tumor of different types of leukemia^13^. miRNA is highly stable in both fresh and stored samples of plasma, making it the most potential blood-based biomarker of diagnostic and prognostic significance^11^. Several studies have shown that there are distinct microRNAs for specific cancer types that differentiate between malignant and its adjacent normal tissue^6^. miR-146a has been reported to play a key role in immune system, inflammation, myeloproliferation and oncogenesis^14^. As an oncogene its up-regulation may alter hematopoiesis leading to the development of leukemia^13,15^. There are few reports available in the literature on the role of miR-146a in ALL. A study reported the up-regulation of miR-146a in ALL samples of children^16^. Another report described its down-regulation in adult AML patient samples^17^. The miR-146a has shown an important role in hematopoiesis, predominantly in T-cell development affecting proliferation and apoptosis of NB4 cells^18^. Some abnormal phenotypes in hematopoietic system were found in a mice deficient with miR-146a that indicates its potential role in hematopoiesis^19^. The overexpression of miR-146a has been reported in stem cell lines including monocytic and lymphocytic lineage after bone marrow transplant^17^. A study presented microarray results of miRNA profiling on bone marrow biopsy and peripheral blood samples of childhood ALL. After validation through qPCR, up-regulation of miR-146a was revealed in bone marrow biopsy but its overexpression in blood samples was not found^20^. A study on 49 bone marrow samples reported miR-146a overexpression in childhood cases of ALL^21^. Most of these studies reported up-regulation of miR-146a in bone marrow samples of ALL. The studies reporting its expression in plasma samples are limiting. The present research was conducted to validate whether miR-146a levels in plasma of ALL patients were overexpressed or otherwise and analyze the plasma miR-146a expression levels after treatment to evaluate its potential as a biomarker for ALL. The altered expression in the current study provides the basis to utilize circulating miR-146 s as a novel predictive marker for diagnosis and prognosis of adult as well as pediatric-ALL.

## Methods

### Patients and clinical samples

Whole blood samples were collected in EDTA tubes from a total of 110 study subjects including patients (*n* = 66), controls (*n* = 24) and follow up cases (*n* = 20). Patients were diagnosed based on morphology, cytogenetic and immunophenotype and characterized according to WHO classification criteria^22^. The patients registered at INMOL hospital, Lahore, Pakistan from January, 2013 to November, 2016 were included in the study. The patients who were newly diagnosed were designated as “cases” and those with any kind of infectious diseases were excluded to determine accurate representation of the study. The cases monitored after four cycles of chemotherapy and nominated as “follow up cases” were also included to evaluate remission status after treatment. Complete remission was considered with bone marrow having less than 5 blasts percentage. The control samples were obtained from healthy subjects as “normal control” that has no malignancy and infectious disease. Patient’s history, clinical features, hematological and other laboratory parameters, date of diagnosis and start of treatment were recorded on a pre-designed performa.

### Plasma isolation

From whole blood, plasma was isolated within 2–4 h after collection by two step centrifugation at 4 °C, first for 5 min at 2500×*g* and second for 2 min at 3500×*g*. The plasma was separated in RNase free 1.5 mL tubes considering recommended procedures for working on cell free miRNA^23^. Then, aliquots (each of 250 µL) were prepared for isolation of miRNA and down-stream process. The prepared aliquots were stored at − 80 °C till further analysis.

### miRNA isolation from plasma

Plasma was thawed on ice and processed to isolate miRNA according to manufacturer’s instruction provided in the miRNeasy serum/plasma kit from Qiagen (Hilden, Germany). The syn-*cel*-miR-39 (synthetic *caenorhabditis elegans* mir-39) from miRNeasy serum/plasma spike-In control (Qiagen, Hilden, Germany) was added to each denatured sample as exogenous control for normalization of sample-to-sample variation during the RNAisolation^24,25^. The elution containing miRNA was stored at − 20 °C without delay for downstream process. The quality and quantity of RNAwas evaluated using NanoDrop by Thermo Scientific (USA). Quantification of RNAincluding miRNA was performed at 260 nm using RNase free water as a blank. The samples with absorbance ≥ 2 were considered for further analysis.

### cDNA synthesis from RNA including miRNA

cDNA was synthesized by reversely transcribed isolated RNA including miRNA using master mix from miScript II RT kit from Qiagen (Hilden, Germany) using the protocols followed by the manufacturer. RNA with concentration of 100 ng (used as a template) was added to the master mix containing nucleic mix (10 × miScript) HiSpec buffer (5 × miScript), reverse transcriptase and RNase free water. Reaction was carried out in a total of 20 µL volume using thermo cycler from BioRad®. Reaction conditions for reverse transcription were first incubation of 60 min at 37 °C followed by second incubation of 5 min at 95 °C. The synthesized cDNA was carefully stored in microtubes at – 20 °C for further use.

### Quantitative real time polymerase chain reaction (qPCR)

miRNA quantification was performed using relative quantitative method of qPCR. SYBR green dye from Qiagen (Hilden, Germany) was used to measure relative quantification of miRNA from plasma using protocols provided with the kit. Primer assays (10 × miScript) were purchased from Qiagen (Hilden, Germany) that contained forward sequences. Universal primer (available with SYBR green PCR kit) was used as a reverse primer for qPCR assays. The sequences of primers and their accession numbers were listed in Supplementary Table S1. Reactions were run in duplicate using real time thermal cycler, CFX96 from BioRad. Reference gene, hsa-mir-16, was used as a normalizer^26^ and NTC (no template control) was run as a negative control in each run to control for contamination. Normalized fold change was calculated using Livak 2^−ΔΔCt^ method^27^ that utilizes ΔCt (Ct_miR-146a_–Ct_miR-16)_ and ΔΔCt (Ct_miR-146a_–Ct_miR-16_)_mean_ of the patients – (Ct_miR-146a_–Ct_miR-16_)_mean_ of the controls.

### Target prediction analysis of miR-146a

The total 3367 targets of miR-146a were retrieved from miRNA target database target scan (release 7.2)^28^. These targets were evaluated based on their target score and those with highest score were analyzed with MetaCore and STRING (Version 10.5) to search for targets related to ALL.

### Statistical analysis

The data was analyzed using statistical tools like GraphPad prism software (version 7) and SPSS for windows (version 21). For validation of the results, Mann Whitney test, ANOVA (analysis of variance) and student’s t test was applied depending upon different variables. Kruskal Wallis analyses was performed to compare multiple groups. The diagnostic value of miR-146a was evaluated through ROC curves produced by MedCalc (version 15.8). The potential of miR-146a as a prognostic factor was determined using Univariate analysis. Non-parametric spearman’s correlation analyses were performed to evaluate clinical significance of miR-146a in ALL. The *P*-values obtained were two-tailed that considered significant at 0.05. Targets of miR-146a were determined through target predicting software, targetScan, and network and pathway was generated using gene ontology (GO) analysis including STRING and MetaCore software.

### Ethical approval and consent to participate

The study was approved by the ethical review board of the University of the Punjab, Lahore, Pakistan with approval No. 284/15. The study design was constructed according to the guidelines of the declaration of Helsinki to conduct research on human beings. The samples were taken after informed consent form all the study participants.

### Consent for publication

The present study does not contain any individual’s personal information.

## Results

### Characteristics of study subjects

Overall 110 study subjects were included in the study comprising of patients (*n* = 66), normal controls (*n* = 24) and follow up cases (*n* = 20). In patients, there were 66 ALL cases of which 31 from B-ALL and 35 T-ALL. The minimum age of ALL patients enrolled was 4 years and maximum 48 years with a mean age of 18.17 years. There were 42 childhood and 24 adult cases of ALL, of which 53 were males and 13 were females. Detailed demographic and clinical characteristics of the patients were listed (Table 1). The number of healthy controls in this study were 24 of which 16 (66.7%) were male and 8 (3.3)% were female. The mean age (years) of normal healthy controls calculated was 23.708 ± 13.788. The minimum age of healthy controls was 4.0 years and maximum age was 47.0 years. There were 14 children and 10 adults in healthy controls used for this study. Other characteristics of normal (healthy controls) including hematological parameters were shown (Table 2).

**Table 1:** Baseline characteristics of participants, demographic and clinical history of the patients enrolled in the study.

Characteristics	Normal healthy controls (n/mean ± SD)	ALL patients (n/mean ± SD)	Follow up cases (n/mean ± SD)
**Total subjects (*n* = 110)**	24	66	20
**Age**			
Childhood	14	42	15
Adult	10	24	5
**Gender**			
Male (*n*)	16	53	17
Female (*n*)	8	13	3
**Age (years)**	20.708 ± 13.788 (4.0–47.0)	18.17 ± 11.29 (4.0–48.0)	21.600 ± 6.6285 (5.0–43.0)
**Patients Characteristics**	**ALL (*n*)**	**B-ALL (*n*)**	**T-ALL (*n*)**
**Type**	66	31	35
**Age**			
Childhood	42	14	28
Adult	24	17	07
**Gender**			
Male	53	22	31
Female	13	09	04
**Risk**			
High	43	28	15
Standard	23	03	20
**Fever**			
Yes	62	28	34
No	04	03	01
**Weakness**			
Yes	40	20	20
No	26	11	15
**Weight loss**			
Yes	14	06	08
No	52	25	27
**Breathlessness**			
Yes	05	03	02
No	61	28	33
**Anorexia**			
Yes	12	05	07
No	54	26	28
**Pallor**			
Yes	54	23	31
No	12	08	04
**Body ache**			
Yes	41	20	21
No	25	11	14
**Cough**			
Yes	14	04	10
No	52	27	25
**Bleeding**			
Yes	18	06	12
No	48	25	23
**Epistaxis**			
Yes	06	01	05
No	60	30	30
**Bruising**			
Yes	02	00	02
No	64	31	33
**Petechia**			
Yes	05	01	04
No	61	30	31
**Vomiting/nausea**			
Yes	16	04	12
No	50	27	23
**Hepatomegaly**			
Yes	30	14	16
No	36	17	19
**Splenomegaly**			
Yes	44	21	23
No	22	10	12
**Lymphadenopathy**			
Yes	35	12	23
No	31	19	12
**BCR-ABL**			
Negative	60	25	35
Positive	06	06	00
**CNS infiltration**			
Yes	02	00	02
No	64	31	33
**Age (years)**	18.17 ± 11.29 (4.00–48.0)	22.64 ± 13.74 (5.0–48.0)	15.22 ± 6.68 (5.00–38.0)
**Height (cm)**	149.93 ± 27.68 (39.0–185)	153.0 ± 21.74 (95.0–181)	147.1 ± 32.10 (39.0–185)
**Weight (kg)**	49.04 ± 19.98 (13.0–100)	52.22 ± 22.50 (16.0–100)	46.22 ± 17.28 (13.0–78.0)
**Hemoglobin (g/dL)**	7.516 ± 2.12 (2.90–11.9)	7.519 ± 2.03 (3.90–11.9)	7.514 ± 2.22 (2.90–11.9)
**RBC’s (10**^**6**^**/µL)**	2.931 ± 0.83 (1.4–5.40)	2.907 ± 0.851 (1.44–5.38)	2.952 ± 0.839 (1.53–5.40)
**WBC’s (10**^**9**^**/L)**	48.93 ± 88.02 (1.40–480)	25.31 ± 25.24 (1.40–89.7)	69.85 ± 115.3 (2.90–480)
**Lymphocytes (%)**	77.36 ± 9.34 (43.5–89.9)	75.53 ± 9.16 (56.4–88.6)	78.98 ± 9.32 (43.5–89.9)
**Neutrophils (%)**	17.86 ± 8.67 (5.10–44.0)	20.22 ± 8.99 (7.10–41.2)	15.78 ± 7.92 (5.10–44.0)
**Platelets (10**^**9**^**/L)**	45.22 ± 41.61 (1.70–224)	49.24 ± 48.19 (7.00–224)	41.66 ± 35.12 (1.70–154)
**PB blasts**	69.72 ± 23.29 (10.0–96.0)	68.50 ± 23.84 (10.0–96.0)	71.23 ± 22.81 (12.0–95.0)

The values are *n* = number and/or mean ± standard deviation (minimum–maximum).

The data tabulated is analyzed by SPSS for windows (version 21).

**Table 2:** Plasma levels of mir-146a and hematological parameters before and after treatment in ALL.

Parameter	Normal (*n* = 24)	Pre-treatment (*n* = 20)	Post-treatment (*n* = 20)	*P*-value
Hb (g/dL)	13.50 ± 0.79	7.24 ± 2.19	11.32 ± 1.316	< 0.0001
RBCs (10^6^/µL)	4.364 ± 0.51	2.917 ± 0.91	3.953 ± 0.44	< 0.0001
HCT (%)	41.31 ± 2.55	29.81 ± 14.60	35.09 ± 4.55	< 0.0001
MCV (fl)	86.95 ± 5.63	80.20 ± 13.28	86.75 ± 4.67	0.0143
MCH (pg)	30.30 ± 2.67	23.87 ± 5.72	30.31 ± 2.62	< 0.0001
MCHC (g/dL)	33.63 ± 1.67	33.76 ± 7.46	34.08 ± 1.89	0.1738
WBCs (10^9^/L)	8.224 ± 1.87	45.51 ± 78.73	9.441 ± 5.51	< 0.0001
Lymphocytes (%)	31.08 ± 8.70	79.76 ± 9.83	27.71 ± 6.15	< 0.0001
Neutrophils (%)	50.3 ± 10.05	16.37 ± 8.24	50.65 ± 11.2	< 0.0001
Mixed cells (%)	18.62 ± 10.42	3.868 ± 2.68	21.79 ± 12.03	< 0.0001
Platelets (10^9^/L)	299.7 ± 81.45	50.01 ± 35.9	289.2 ± 141.2	< 0.0001
Plasma mir-146a (Fold change)	1.365 ± 1.03	34.71 ± 15.26	0.9946 ± 0.83	< 0.0001

The values are mean ± SD (standard deviation). *P*-values are significant at < 0.05.

### Circulating miR-146a is up-regulated in ALL

**Figure 1:**
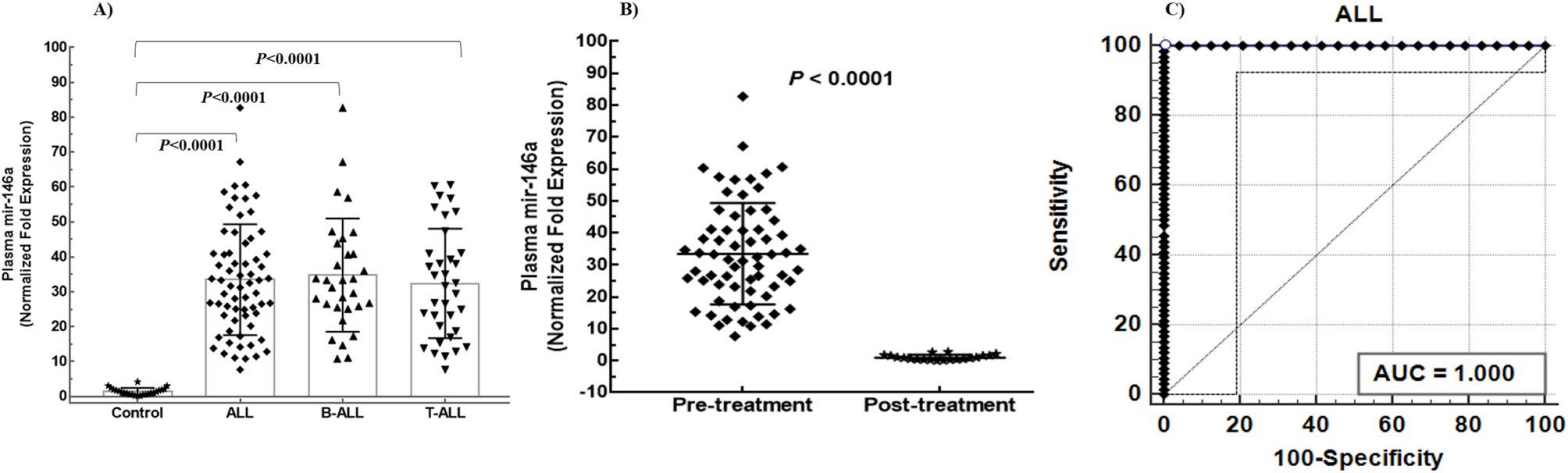
Normalized fold expression of plasma mir-146a. (**A**) Relative quantification of plasma mir-146a in patients with ALL (n = 84), B-ALL (n = 48), T-ALL (n = 39), and healthy controls (n = 25). The y-axis denotes relative expression of plasma mir-146a normalized to mir-16 as a reference gene. (**B**) The levels of plasma mir-146a significantly reduced after treatment in patients of ALL (P < 0.0001). (**C**) Receiver operating curve (ROC) analyses for ALL with an AUC of 1.00 representing 100% sensitivity and 100% specificity.

**Figure 2:**
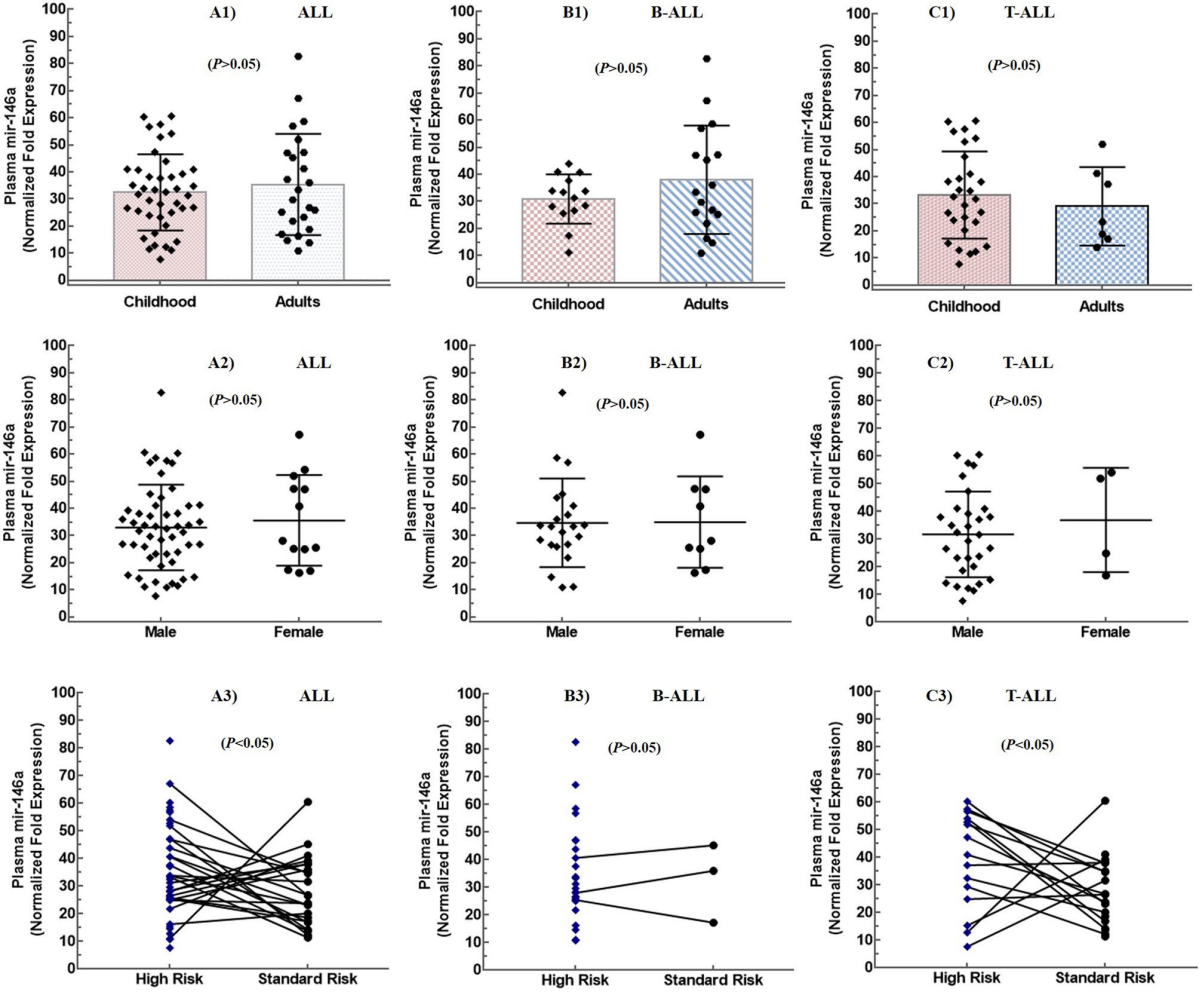
Association of plasma mir-146a expression with age, gender and risk status. Normalized fold expression of mir-146a in childhood and adult patients in (**A1**) ALL, (**B1**) B-ALL, (**C1**) T-ALL. Scatter plots represented no significant difference in mir-146a expression levels in childhood compared with adults. There were no gender differences in mir-146a expression in plasma of (**A2**) ALL, (**B2**) B-ALL, (**C2**) T-ALL. Fold expression of mir-146a was significantly increased in high risk groups compared with standard risk in (**A3**) ALL and (**C3**) T-ALL, however, no significant difference was observed in (**B3**) B-ALL.

**Figure 3:**
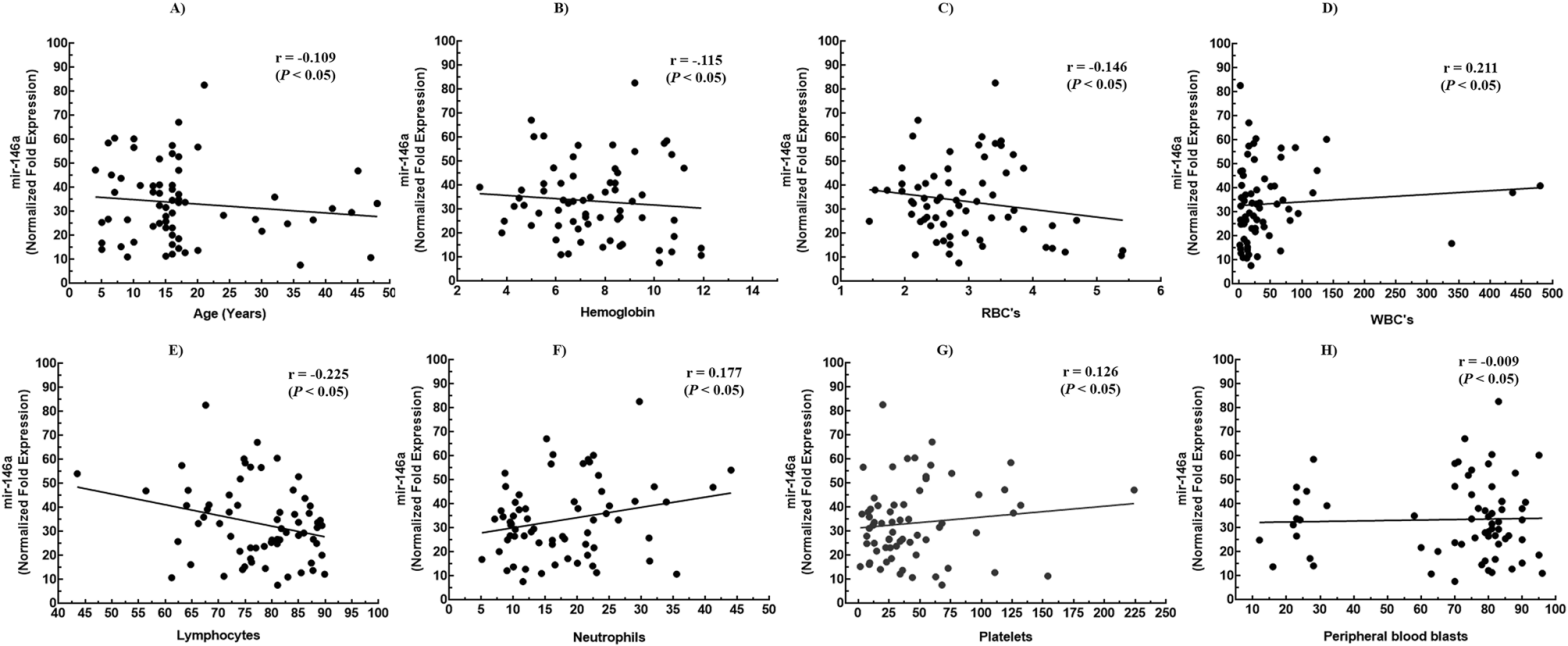
Correlation analysis of plasma mir-146a in ALL. (**A**) Age, (**B**) Hemoglobin, (**C**) RBC’s, (**D**) WBC’s, (**E**) Lymphocytes, (**F**) Neutrophils, (**G**) Platelets, (**H**) Peripheral blood blasts. The expression levels of mir-146a in plasma of ALL patients were not significantly correlated with all the parameters.

To explore potential role of circulating miR-146a in the development of ALL, we determined its normalized fold expression in plasma samples of patients and controls by relative quantification using real time PCR. Fold change calculated by 2^−ΔΔCt^ method revealed significant up-regulation of miR-146a in patients of ALL (33.46 ± 15.84) B-ALL (34.74 ± 16.19) and T-ALL (32.22 ± 15.66), compared with controls (1.36 ± 1.03) with a *P*-value of < 0.0001 (Fig. 1A). However, there was no significant difference of expression in ALL subtype, and childhood and adults in ALL, B-ALL, and T-ALL (*P* > 0.05) (Fig. 2A1–C1). Moreover, no gender differences were observed in all the subtypes of ALL (*P* > 0.05) (Fig. 2A2–C2). However, when categorized according to WHO risk classification, patients of ALL with high risk showed higher mean expression levels of mir-146a, compared with standard risk patients (*P* = 0.0469) (Fig. 2A3). The categorization based on risk also showed statistically significant differences in T-ALL (Fig. 2C3) although these differences were not significant in B-ALL (Fig. 2B3). We found that levels of plasma miR-146a in ALL patients were independent of subtype, age and gender. Furthermore, correlation analysis between age and miR-146a was insignificant (*P* < 0.05) that confirmed its importance as a diagnostic marker for childhood as well as adult ALL (Fig. 3A).

### Plasma miR-146a levels are independent of hematological parameters

A remarkable feature of a diagnostic marker is that it should be independent of clinical relevance and show no evident change with clinical and hematological parameters. To identify this aspect, correlation analysis was performed between miR-146a levels and hematological parameters. We could not detect any significant correlation with hemoglobin, RBC’s, WBC’s, lymphocytes, neutrophils, platelets and peripheral blood blasts (*P* < 0.05) (Fig. 3B–H). The insignificant correlation proved that miR-146a levels in plasma were not affected by hematological parameters and could not influence its significance to use as a diagnostic marker for ALL.

### Plasma miR-146a as a diagnostic marker for ALL

To evaluate diagnostic potential of plasma miR-146a in ALL, ROC (receiver operating curve) analyses were performed that showed area under curve (AUC) of 1 for ALL (95% CI 0.960 to 1.00) having 100% sensitivity, 100% specificity (*P* < 0.0001) (Fig. 1C). For B- and T-ALL, AUC of 1 was observed (95% CI 0.935 to 1.00) showing 100% sensitivity and 100% specificity (*P* < 0.0001).

### Plasma miR-146a as a prognostic marker for ALL

**Figure 4:**
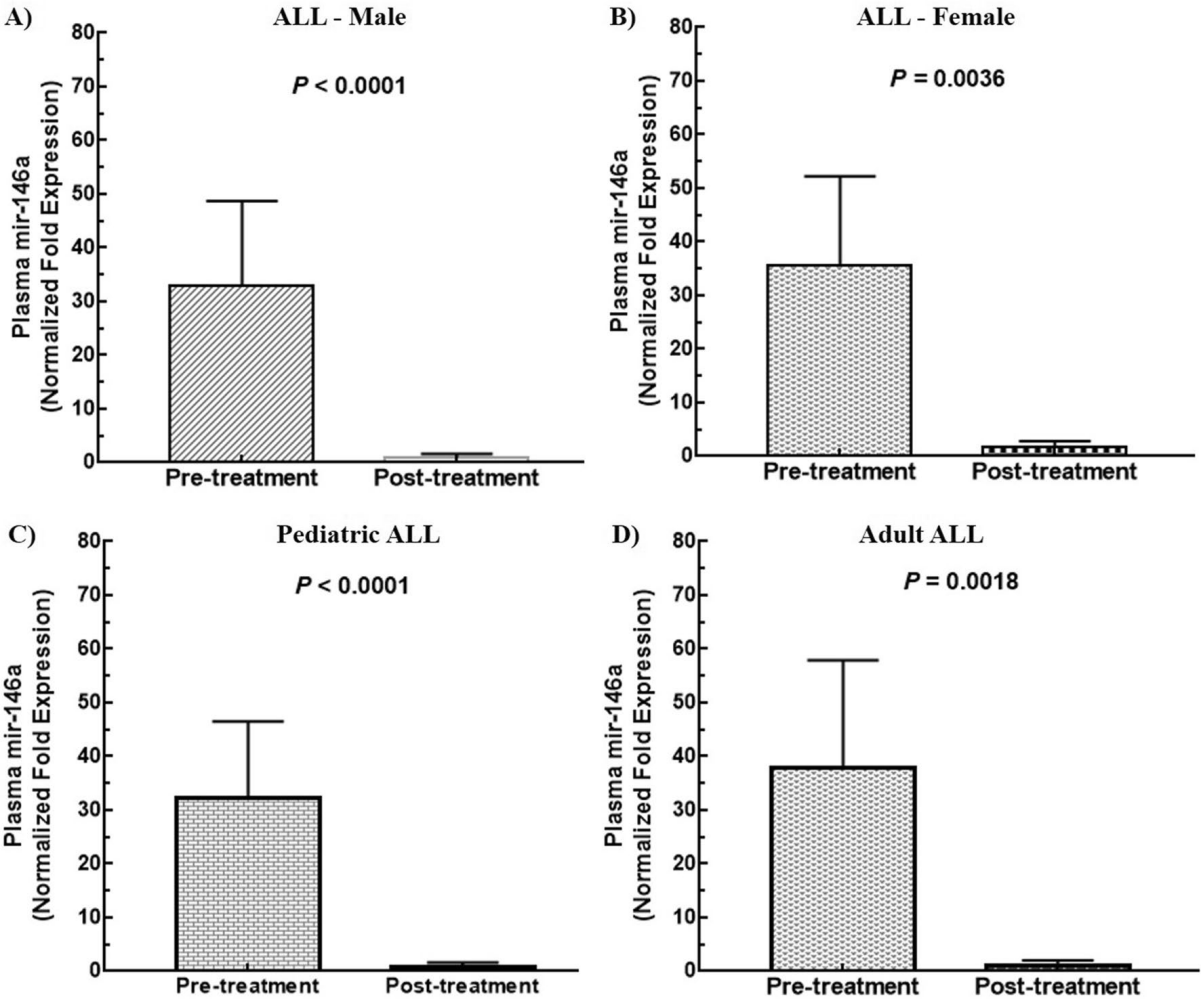
Comparison of plasma levels mir-146a expression before and after treatment in ALL. (**A**) males (**B**) females (**C**) pediatric ALL (**D**) adult ALL. Mean fold expression of plasma mir-146a significantly reduced after treatment in all the above-mentioned cases (*P* < 0.05).

To evaluate prognostic impact of miR-146a in plasma of ALL, expression levels were analyzed before and after complete rounds of chemotherapeutic treatment in paired samples. It was noted that mean expression of miR-146a before chemotherapy was 33.46 ± 15.84, 34.74 ± 16.19 and 32.33 ± 15.66 in patients of ALL, B-ALL and T-ALL, respectively. Interestingly, these levels significantly decreased after treatment with a *P* value of < 0.0001 (Fig. 1B). Mean fold expression in male patients of ALL was 32.95 ± 15.74 that reduced to 0.8865 ± 0.754 showing significant difference (*P* < 0.0001) (Fig. 4A). Similarly, plasma miR-146a expression in females with ALL was 35.55 ± 16.68 that decreased to 1.61 ± 1.196 (*P* = 0.0036) (Fig. 4B). Moreover, miR-146a mean levels in plasma of pediatric and adult samples were 32.4 ± 14.06 and 35.33 ± 18.72, that reduced to 0.8486 ± 0.7688 and 1.337 ± 0.9601, respectively, (*P* < 0.0001) (Fig. 4C,D). Furthermore, miR-146a expression levels were significantly lowered in after chemotherapy in childhood B-ALL (*P* = 0.0167), adult B-ALL (*P* = 0.0018), childhood T-ALL (*P* < 0.0001) and adult T-ALL (*P* = 0.0167). In addition, we analyzed pre- and post-treated levels of hematological parameters along with miR-146a expression that also showed significant decrease after treatment (Table 2).

### Potential predictive targets of miR-146a

**Figure 5:**
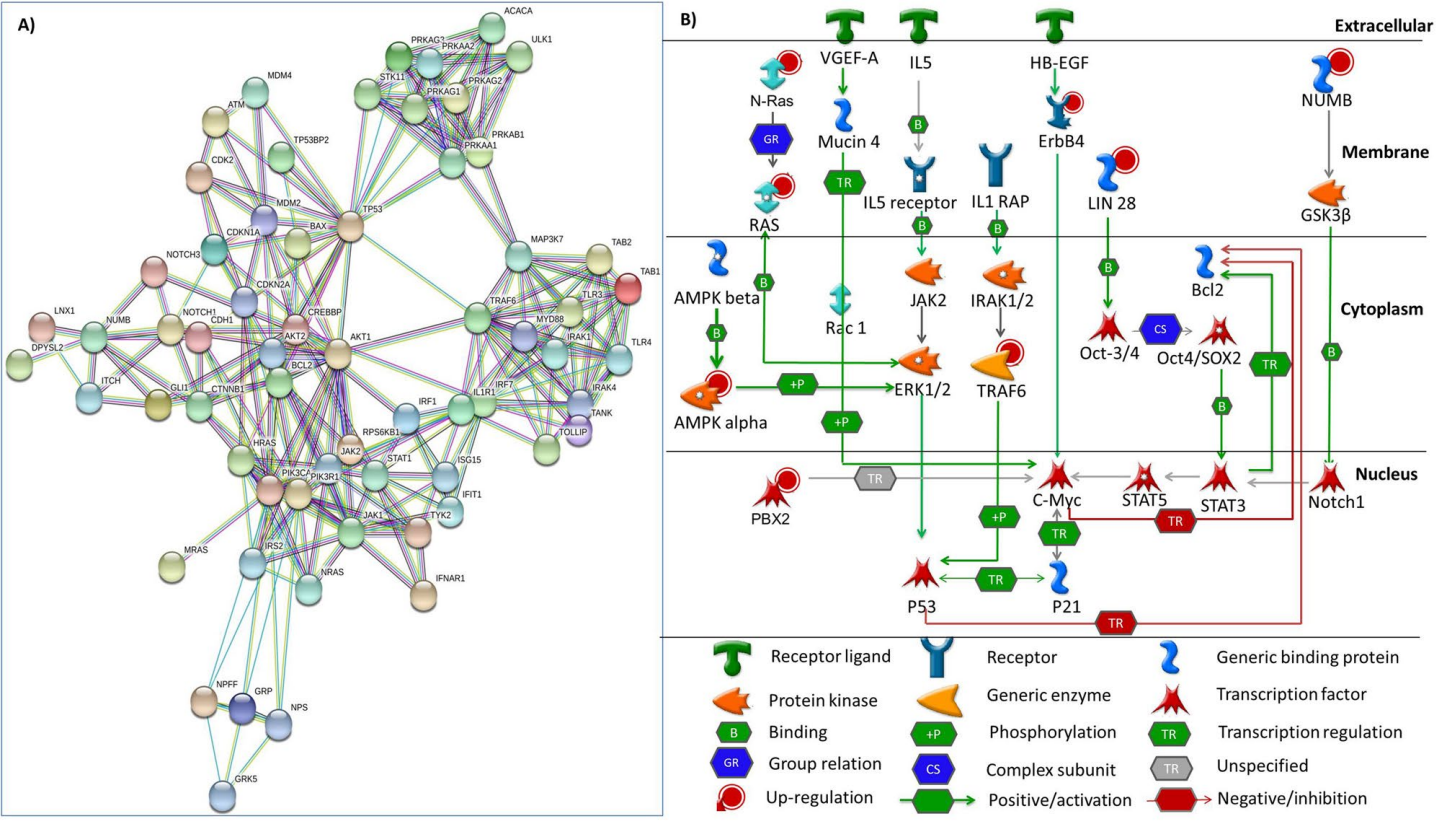
Target prediction analysis of mir-146a. (**A**) Network analysis of mir-146a predicted targets by STRING. The targets with score > 0.9 and *P* < 1.0e−16 were selected to achieve highest confidence (**B**) Pathway analysis performed using MetaCore showed up-regulated targets genes of mir-146a highlighted with round shape (red). The thickness of lines showed the extent of interaction with the target molecules.

miR-146a targets a number of tumor suppressor genes that have shown their role in the pathogenesis of ALL. We first, selected targets of highest score (≥ 0.90) through targetScan and then analyzed these targets by STRING as shown (Fig. 5A). The genes predicted in the network were involved in cell cycle, proliferation, cell growth, development, DNA replication and repair mechanism and cell death. Further, we constructed a hypothetical pathway of these targets on MetaCore software for predicting novel targets for miR-146a (Fig. 5B). The round shape highlighted with red color showed the up-regulated targets that include *N-RAS*, *RAS*, *AMPK-alpha*, *PBX2*, *ErbB4*, *TRAF6*, *LIN28* and *NUMB*. These are involved in the regulation of gene expression by transducing signals from cytoplasm to the nucleus. *N-RAS* and *RAS* present in the cell membrane are transported into the cytoplasm and then bind to *ERK1/2*. In the cytoplasm, *AMPK-alpha* subunit is phosphorylated and binds with *ERK1/2*. Subsequently, *ERK1/2* and *TRAF6* activates *P53*, a tumor suppressor gene in the nucleus that negatively regulates *Bcl2* causing reduced apoptosis leading to uncontrolled proliferation of leukemic cells. The oncogene, *C-Myc* has an important role in cell growth, proliferation and apoptosis. *LIN28* binds with *Oct3/4-SOX2* complex and activates *C-Myc*, involving *STAT5* and *STAT3*. Similarly, *PBX2*, *ErbB4* and *NUMB* activate *C-Myc* by activating *Notch1* pathway. Thus, by regulating tumor suppressor and oncogenes, aforementioned targets caused increased proliferation, abnormal cell growth and reduced cell death ultimately leading to the development of ALL.

## Discussion

There has been an emerging interest in miRNA to explore its potential as a predictive marker for diagnosis and prognosis of several hematological diseases including ALL. In the previous studies, miR-146a has shown a major role in the hematopoiesis, predominantly in T-cell development influencing proliferation and apoptosis of NB4 cells^18^. Another study developed a mice deficient with miR-146a and found some abnormal phenotypes in hematopoietic system indicating its potential role in hematopoiesis^19^. Aberrant miR-146a expression has been studied in ALL. A single report described decreased expression of miR-146a in blood and bone marrow of children with T-ALL^29^. All other reports showed up-regulation of miR-146a in bone marrow. We performed our experiment on plasma samples isolated from whole blood to determine whether miR-146a levels in plasma were overexpressed or otherwise.

In the current work, we, first, found up-regulation of miR-146a in plasma samples of childhood and adult cases of ALL, compared with healthy subjects as normal controls. Previously, overexpression of miR-146a has been reported in stem cell lines including monocytic and lymphocytic lineage after bone marrow transplant^17^. miR-146a has shown oncogenic role in childhood ALL as it has shown up-regulation in pediatric samples of ALL^16^. A study presented microarray results of miRNA profiling on bone marrow biopsy and peripheral blood samples of childhood ALL. After validation through qPCR revealed up-regulation of miR-146a in bone marrow biopsy but could not find its overexpression in blood samples^30^. A study on 49 bone marrow samples reported miR-146a overexpression in childhood cases of ALL^21^. We were able to determine its expression both in children as well as adults with ALL and its subtypes including B-and T-cell. Another study reported down-regulation of miR-146a expression in adult AML patients^17^ that enhanced disease status by regulating *TRAF6* involved in NF-κB pathway^31^.

Further, we analyzed clinical relevance of miR-146a with hematological parameters that showed no significant correlation with WBC’s, RBC’s, hemoglobin, platelets, neutrophils and lymphocytes. On the basis of these results, it may be suggested that miR-146a levels were not affected by the clinical features. These results are in agreement with the previous finding that also reported no clinical association with WBC’s, hemoglobin and platelets count in ALL^32^.

The most striking feature of the current research was determination of miR-146a expression levels after treatment in paired samples of ALL. We were able to find significant reduction in miR-146a expression after chemotherapy demonstrating its prognostic significance in plasma samples of pediatrics and adults. Similar effects of treatment were reported on bone marrow samples by Duyu et al., who described post-treatment decrease in miR-146a expression in childhood ALL^30^. Association of poor survival with overexpression of miR-146a in adults of ALL has been described^33^. Moreover, favorable treatment outcome in ALL patients was related to miR-146a down-regulation^34^. The significant reduction in expression of miR-146a in pre- and post-treatment plasma samples indicated its prognostic significance and it was suggested that plasma miR-146a has a potential to predict treatment response and can be utilized as a prognostic marker of pediatric and adult ALL.

miRNA may act as an oncogenic or tumor suppressor based on the target gene regulated by miRNA^13^. miR-146a is a dual nature miRNA showing both tumor suppressor and oncogenic properties in different types of leukemia. Studies reported its role as a tumor suppressor by regulating *STAT1*, an apoptotic factor, and *Bcl-XL*, an antiapoptotic factor, promoting apoptosis in jurkat cells in ALL cell lines^35^. It has been reported to target *TRAF* and *IRAK6* that are regulatory genes of immune response^36^. Previously, the targets for mir-146a i.e., *TRAF6*, *IRAK1*^31^, and *IRAK2*^37^, were reported to be involved in immune system. We found the role of these targets in ALL that may serve as potential targets for treatment of ALL.

## Conclusion

We concluded that miR-146a up-regulation in plasma may be utilized as a novel, non-invasive marker for diagnosis of ALL in childhood and adults. Furthermore, treatment response may be monitored through altered miR-146a expression levels that may improve prognosis of pediatric and adult ALL. The non-invasive diagnosis and prognosis may overcome poor overall survival of patients with ALL and good treatment outcome. miR-146a target prediction may also pave a way to understand the mechanisms in the regulation of gene expression establishing more effective targets for ALL therapy. Therefore, plasma miR-146a may be employed as a promising marker for diagnosis, prognosis and treatment of adult as well as pediatric ALL.

## Supplementary Information

Supplementary Information.

## Data Availability

The datasets used and/or analyzed during the current study are available from the corresponding author on reasonable request.
