# Supplementary material for: Circulating miR-146a expression as a non-invasive predictive biomarker for acute lymphoblastic leukemia
Source: Sci Rep. 2021 Nov 23;11:22783. doi: 10.1038/s41598-021-02257-4 (PMC8611079; doi:10.1038/s41598-021-02257-4)
Supplement: Supplementary file 1 — Supplementary Information. [file 41598_2021_2257_MOESM1_ESM.pdf]

Title

Circulating miR-146a expression as a non-invasive predictive biomarker for acute lymphoblastic leukemia

Running Title

Circulating miR-146a as non-invasive biomarker for ALL

Authors

Samiah Shahid[1,2*], Wajeehah Shahid[3], Jawaria Shaheen[1], M. Waheed Akhtar[1,4], Saima Sadaf[1]

Authors' Affiliation

[1]School of Biochemistry and Biotechnology, University of the Punjab, Lahore-54590, Pakistan

[2] Institute of Molecular Biology & Biotechnology, The University of Lahore, Lahore, Pakistan

[3] Department of Physics, The University of Lahore, Lahore, Pakistan.

[4]School of Biological Sciences, University of the Punjab, Lahore-54590, Pakistan

[*] **Corresponding Author**

Dr. Samiah Shahid
Assistant Professor
Institute of Molecular Biology & Biotechnology
The University of Lahore, Pakistan
samiah.shahid@imbb.uol.edu.pk
Contact: 00923314578806

Supplementary material

Table S1: Primers of microRNAs with their mature sequence and accession number

MicroRNA/snRNA	Mature Sequence	Accession number
hsa-miR-146a	5′-UGAGAACUGAAUUCCAUGGGUU-3′	MIMAT0000449
hsa-miR-16	5′-UAGCAGCACGUAAAUAUUGGCG-3′	MIMAT0000069
